# Negative Regulation of Hepatitis C Virus Specific Immunity Is Highly Heterogeneous and Modulated by Pegylated Interferon-Alpha/Ribavirin Therapy

**DOI:** 10.1371/journal.pone.0049389

**Published:** 2012-11-08

**Authors:** Mark A. A. Claassen, Robert J. de Knegt, Duygu Turgut, Zwier M. A. Groothuismink, Harry L. A. Janssen, André Boonstra

**Affiliations:** Department of Gastroenterology and Hepatology, Erasmus MC – University Medical Center Rotterdam, Rotterdam, The Netherlands; Duke University, United States of America

## Abstract

Specific inhibitory mechanisms suppress the T-cell response against the hepatitis C virus (HCV) in chronically infected patients. However, the relative importance of suppression by IL-10, TGF-β and regulatory T-cells and the impact of pegylated interferon-alpha and ribavirin (PegIFN-α/ribavirin) therapy on these inhibitory mechanisms are still unclear. We revealed that coregulation of the HCV-specific T-cell responses in blood of 43 chronic HCV patients showed a highly heterogeneous pattern before, during and after PegIFN-α/ribavirin. Prior to treatment, IL-10 mediated suppression of HCV-specific IFN-γ production in therapy-naive chronic HCV patients was associated with higher HCV-RNA loads, which suggests that protective antiviral immunity is controlled by IL-10. In addition, as a consequence of PegIFN-α/ribavirin therapy, negative regulation of especially HCV-specific IFN-γ production by TGF-β and IL-10 changed dramatically. Our findings emphasize the importance of negative regulation for the dysfunctional HCV-specific immunity, which should be considered in the design of future immunomodulatory therapies.

## Introduction

In the vast majority of patients, the hepatitis C virus (HCV) causes chronic infection with viral replication primarily in the liver. The inability to mount strong, broadly targeted and lasting CD4^+^ and CD8^+^ T-cell responses against HCV is considered crucial for the development and maintenance of this persistent infection (reviewed in [1], [2], [3]). Several host and viral mechanisms have been proposed to contribute to this deficient HCV-specific T-cell response, including viral escape, impaired antigen presentation, anergy, exhaustion of the T-cell response mediated via inhibitory receptors such as PD-1 and Tim-3 [1], [2], and active suppression of virus-specific T-cell responses by regulatory T-cells (Treg) expressing the transcription factor FoxP3 or by the immunosuppressive cytokines IL-10 or TGF-β [4]. A recent paper by Raziorrouh and colleagues suggests that inhibition of IL-10 and TGF-β simultaneously is the most promising condition to restore HCV-specific T-cell responses [5].

We and others provided evidence for increased numbers of FoxP3^+^Treg in the liver of chronic HCV patients [6], [7], [8], [9]. Treg in blood have been shown to suppress both HCV-specific proliferation and IFN-γ production by CD4^+^ and CD8^+^ T-cells [10], [11], [12], [13], [14]. Moreover, it has been suggested that Treg, at least partially, control chronic liver inflammation, with higher Treg suppressive capacity in patients with lower serum alanine transaminase (ALT) levels [11]. Other studies have shown that blocking IL-10 or TGF-β can enhance HCV-specific T-cell proliferation and IFN-γ production [15], [16], [17], [18], [19]. In addition, serum IL-10 and TGF-β levels were enhanced in HCV-infected patients as compared to control individuals, and augmented production of these inhibitory cytokines by monocytes and T-cells has been described for HCV-infected patients [15], [17], [18], [19], [20], [21], [22].

Despite overwhelming evidence on the importance of IL-10, TGF-β and Treg in controlling immunity to HCV, little information is available on the coregulation of these regulatory mechanisms by assessing whether multiple pathways simultaneously contribute to impairment of HCV-specific immunity. Also, the modulation of immunoregulatory pathways by antiviral therapy of chronic HCV patients has received little attention, but is highly relevant since it is still under debate whether pegylated interferon-α and ribavirin (PegIFN-α/ribavirin) therapy restores the dysfunctional HCV-specific T-cell response or not [23].

In this study, we therefore investigated the relative contribution of suppression by IL-10, TGF-β and Treg to dysfunctional HCV-specific immunity in peripheral blood from chronic HCV patients. In addition, we studied whether this regulation had clinical implications for these patients. Finally, a detailed examination of coregulation by multiple inhibitory mechanisms was conducted during and after PegIFN-α/ribavirin therapy. Our findings reveal that regulation of the HCV-specific T-cell response in chronic HCV patients is highly heterogeneous, as individuals show distinct patterns of suppression by IL-10, TGF-β or Treg. However, relatively high viral loads were observed in therapy-naive chronic HCV patients showing pronounced IL-10 driven suppression of HCV-specific IFN-γ production. In addition, irrespective of viral response to therapy, negative regulation of especially HCV-specific IFN-γ production by TGF-β and IL-10 changed dramatically as a consequence of PegIFN-α/ribavirin therapy.

## Materials and Methods

### Patients, Controls and Antiviral Therapy

Forty-three chronic HCV infected patients were included (Table 1 and Figure S1 for patient details). Patients were excluded in case of decompensated liver disease, HBsAg positivity, or HIV co-infection. Diagnostic core biopsy samples from 36 of 43 patients, obtained within 3 months prior to start of therapy, were assessed for fibrosis stage by an experienced liver pathologist (Metavir score). Twenty-one patients had never received therapy for HCV before (therapy-naive) and 22 were nonresponders to previous IFN-α and ribavirin therapy (therapy-experienced). A cross-sectional immunological study was performed in all 43 patients. In addition, a longitudinal study followed, in which the 21 therapy-naive patients were investigated at week 4 and 12 during PegIFN-α/ribavirin therapy, and during the follow-up (FU) period at week 4 and 24 after ending treatment. Therapy consisted of twice daily orally administered ribavirin (<65 kg;800 mg/day, 65–80 kg;1000 mg/day, 81–105 kg;1200 mg, >105 kg;1400 mg, Rebetol®, Schering-Plough now MSD, Houten, the Netherlands) and weekly subcutaneous injections with pegylated interferon-α-2b (1.5 µg/kg, PegIntron®, Schering-Plough now MSD). Furthermore, 18 age and sex matched healthy control subjects were included. The institutional ethical review board of the Erasmus MC, Rotterdam approved the clinical protocols, and written informed consent was obtained from all individuals prior to their inclusion. The study was conducted according to the principles expressed in the Declaration of Helsinki.

**Table 1:** Patient characteristics.

							Therapy	Sustained	SNP	SNP	SNP		
Studynumber	Sex	Age	Liverfibrosis	Genotype	HCV RNA	ALT	naïve	Viral	IL-10	TGF-β	IL-28B	IP-10	
	(M/F)	(years)	(Metavir)		(IU/mL)	(U/L)		Response				(pg/ml)	
1	F	57	3	1	1.1×10^7^	150	Yes	Yes	GG	GG	TC	698	
2	F	37	n.d.	3	3.2×10^3^	79	Yes	Yes	GA	GG	TT	206	
3	M	54	1	1	2.7×10^7^	49	Yes	No	GA	GG	TC	99	
4	F	27	1	1	3.7×10^2^	34	Yes	Yes	GG	GG	**CC**	153	
5	M	48	2	3	1.1×10^5^	146	Yes	Yes	**AA**	GG	**CC**	108	
6	M	51	2	1	3.1×10^6^	46	Yes	No	GA	GG	TC	89	
7	M	52	2	3	1.5×10^5^	41	Yes	Yes	**AA**	GG	TC	113	
8	M	34	2	1	3.8×10^6^	103	Yes	Yes	**AA**	n.a.	**CC**	237	
9	M	56	2	1	6.5×10^6^	157	Yes	No	GA	GG	TT	596	
10	F	57	4	1	7.7×10^5^	17	Yes	No	GA	GG	TC	257	
11	M	45	2	1	4.5×10^6^	55	Yes	Yes	GA	**GC**	**CC**	922	
12	M	47	1	1	4.5×10^5^	227	Yes	Yes	GA	GG	TC	319	
13	F	41	1	3	8.6×10^5^	46	Yes	Yes	GA	GG	TC	372	
14	M	60	3	3	3.2×10^6^	164	Yes	Yes	n.a.	**GC**	**CC**	141	
15	M	40	2	1	2.2×10^4^	58	Yes	No	GA	GG	**CC**	396	
16	F	40	3	1	3.1×10^5^	120	Yes	No	GA	GG	TT	590	
17	M	45	n.d.	1	1.1×10^7^	36	Yes	No	GA	GG	TC	1248	
18	F	58	4	1	1.6×10^5^	179	Yes	Yes	**AA**	GG	TC	653	
19	M	57	0	1	7.2×10^6^	48	Yes	No	**AA**	GG	TC	153	
20	M	42	1	1	1.5×10^6^	65	Yes	Yes	**AA**	**GC**	TC	377	
21	F	42	n.d.	1	3.3×10^6^	107	Yes	Yes	GG	GG	TC	993	
22	M	47	1	1	5.3×10^5^	144	No	n.a.	GA	**GC**	TC	277	
23	M	43	4	1	3.4×10^5^	89	No	n.a.	GA	GG	TC	233	
24	F	50	3	1	6.1×10^5^	111	No	n.a.	**AA**	GG	TT	581	
25	F	55	0	1	5.5×10^5^	59	No	n.a.	n.a.	n.a.	TC	875	
26	M	44	0	1	6.7×10^5^	33	No	n.a.	GG	GG	TC	127	
27	F	48	1	1	6.0×10^5^	61	No	n.a.	GA	GG	TT	622	
28	M	45	3	1	1.6×10^6^	57	No	n.a.	**AA**	GG	TT	289	
29	M	49	0	1	3.9×10^5^	48	No	n.a.	GA	GG	TC	469	
30	M	36	4	1	9.3×10^5^	98	No	n.a.	GA	GG	TT	818	
31	M	48	3	1	2.6×10^6^	122	No	n.a.	GG	GG	TC	208	
32	M	41	4	1	1.9×10^5^	120	No	n.a.	GA	GG	TC	357	
33	M	44	n.d.	1	6.2×10^2^	44	No	n.a.	**AA**	GG	**CC**	54	
34	F	53	n.d.	1	2.4×10^6^	77	No	n.a.	**AA**	GG	**CC**	151	
35	M	43	2	1	6.1×10^5^	224	No	n.a.	GA	GG	TC	>1500	
36	M	50	n.d.	1	1.8×10^5^	55	No	n.a.	GA	GG	TC	266	
37	M	41	2	1	3.4×10^5^	67	No	n.a.	GA	GG	TT	369	
38	M	59	3	1	9.3×10^5^	84	No	n.a.	GG	**GC**	**CC**	308	
39	M	39	2	1	8.3×10^5^	53	No	n.a.	**AA**	GG	TC	286	
40	F	46	0	1	5.2×10^4^	74	No	n.a.	**AA**	GG	TC	>1500	
41	M	41	2	1	4.5×10^5^	57	No	n.a.	**AA**	GG	TT	680	
42	M	44	n.d.	4	2.3×10^5^	33	No	n.a.	GA	GG	TC	140	
43	M	48	0	1	2.4×10^6^	56	No	n.a.	GA	GG	TC	359	

Abbreviations: n.d., not determined within 3 months before start of therapy; n.a., not applicable.

### *In vitro* Quantification of HCV-specific T-cell Proliferation and IFN-γ Production

Peripheral blood mononuclear cells (PBMC) were isolated from venous blood by ficoll separation (Ficoll-Paque™ plus, Amersham, Buckinghamshire, UK) and immediately cultured in quadruplets in 96-well round-bottom plates (2x10^5^ cells in 200 µL). Cells were always stimulated with the same pool of overlapping peptides (1 µg/mL per individual peptide; in total 361 individual peptides spanning the core, NS3, NS4, NS5a and NS5b HCV genome; clone J4, genotype 1b; BEI Resources, Manassas, USA), anti-CD3 antibody (1 µg/mL; OKT-3; Janssen-Cilag, Tilburg, the Netherlands), CMV antigens (34 µg/mL; AD-169; Microbix, Toronto, Canada) or no stimulus. DMSO concentrations in all cultures did not exceed 0.5%, which is the concentration generally considered to be acceptable [24]. Culture medium was RPMI 1640 supplemented with L-glutamin, Penicillin-Streptomycin, HEPES, and 5% human serum (all from Lonza, Verviers, Belgium), anti-CD28 (1 µg/mL; CD28.2; eBioscience, San Diego, USA) and anti-CD49d antibody (1 µg/mL; 9F10; eBioscience). After culturing for 3 days, 100 µL supernatant was collected from each well, stored at −80°C, and replaced by fresh culture medium. Supernatants were not pooled and IFN-γ levels were determined in quadruplets in these supernatants by ELISA (Ready Set Go; eBioscience). After stimulation for 5 days, cells were pulsed for 16h with [^3^H]-thymidine (0.5 µCi/well; Amersham, Little Chalfont, UK). Proliferation was determined as counts per minute (cpm) by liquid scintillation. For HCV-specific T-cell proliferation and IFN-γ assays, results were considered positive when more than 500 counts or 100 pg/mL were detected above background, which corresponded to 3 SD above any response observed in healthy controls (data not shown).

### Blocking and Depletion Experiments

Neutralizing antibodies against the IL-10 receptor (IL-10R, 5 µg/mL; 3F9; Biolegend, San Diego, USA), or TGF-β (5 µg/mL; 1D11, kindly provided by Dr. Boon, Bioceros, Utrecht, the Netherlands) were added to the cultures and HCV-specific assays were performed as described above. In addition, assays were performed with PBMC from which CD25^hi^ cells were depleted using CD25 magnetic beads (Miltenyi Biotech, Bergisch Gladbach, Germany). This strategy allowed depletion of at least 80% of CD4^+^CD25^+^FoxP3^+^ Treg (data not shown). HCV-specific responses were considered regulated by Treg, TGF-β or IL-10 when proliferation or IFN-γ levels increased by at least 500 cpm or 100 pg/mL, respectively.

### *In vitro* Quantification of Circulating IP-10, IL-10, TGF-β and Treg levels

Serum IP-10, IL-10 and latent TGF-β levels were determined by ELISA (IP-10: Quantikine Kit, IL-10; Quantikine-HS-kit, RnD, Minneapolis, USA and TGF-β; Ready–Set-Go; eBioscience). To determine the frequency and phenotype of circulating FoxP3^+^Treg cells, venous blood samples were fixed and erythrocytes lysed using FixPerm™ reagent (eBioscience). Samples were stained with antibodies against CD25-PE-Cy7 (2A3; BD), FoxP3-APC (PCH101; eBioscience), CD4-APC-H7 (SK3; BD) and CD3-AmCyan (SK7; BD). Cell acquisition was performed on a FACSCanto II flowcytometer (BD), and analyzed using FacsDiva™ software (BD). For analysis, the gating strategy used to identify FoxP3^+^Treg cells was identical as used in our previous studies [9], [25]. Furthermore, all CD4+CD25^+^FoxP3^+^Treg did not express CD127 (data not shown). The gates were set on the basis of isotype antibody controls, where appropriate.

### Virological Assessment

Serum HCV-RNA levels were determined by quantitative PCR (Cobas® Ampliprep/Cobas® TaqMan® HCV test (limit of detection <15 IU/mL, Roche Diagnostics, the Netherlands). HCV genotypes were determined by an in-house developed sequence analysis assay (Department of Virology, Erasmus MC).

### IL-10 and TGF-β Genotype Determination

The IL28B SNP rs12979860, IL10 SNP rs1800896 and TGF-β SNP rs1800471 variants were determined using competitive allele-specific PCR (KASP; kBioscience ***Hoddesdon***, UK).

### Data Analysis

Data are depicted as mean ±1 SD and unpaired data were compared using Student’s t-test or the Mann Whitney U test for nonparametric data. Correlations between immunological and clinical parameters were calculated using the Spearman correlation test. SPSS 17.0 for Windows (SPSS, Chicago, USA) was used for these analyses. All p-values were two-tailed and considered significant if <0.05.

## Results

### HCV-specific T-cell Proliferation and IFN-γ Production are Weak or Undetectable in Therapy-naive and PegIFN-α/ribavirin Therapy-experienced Chronic HCV Patients

**Figure 1:**
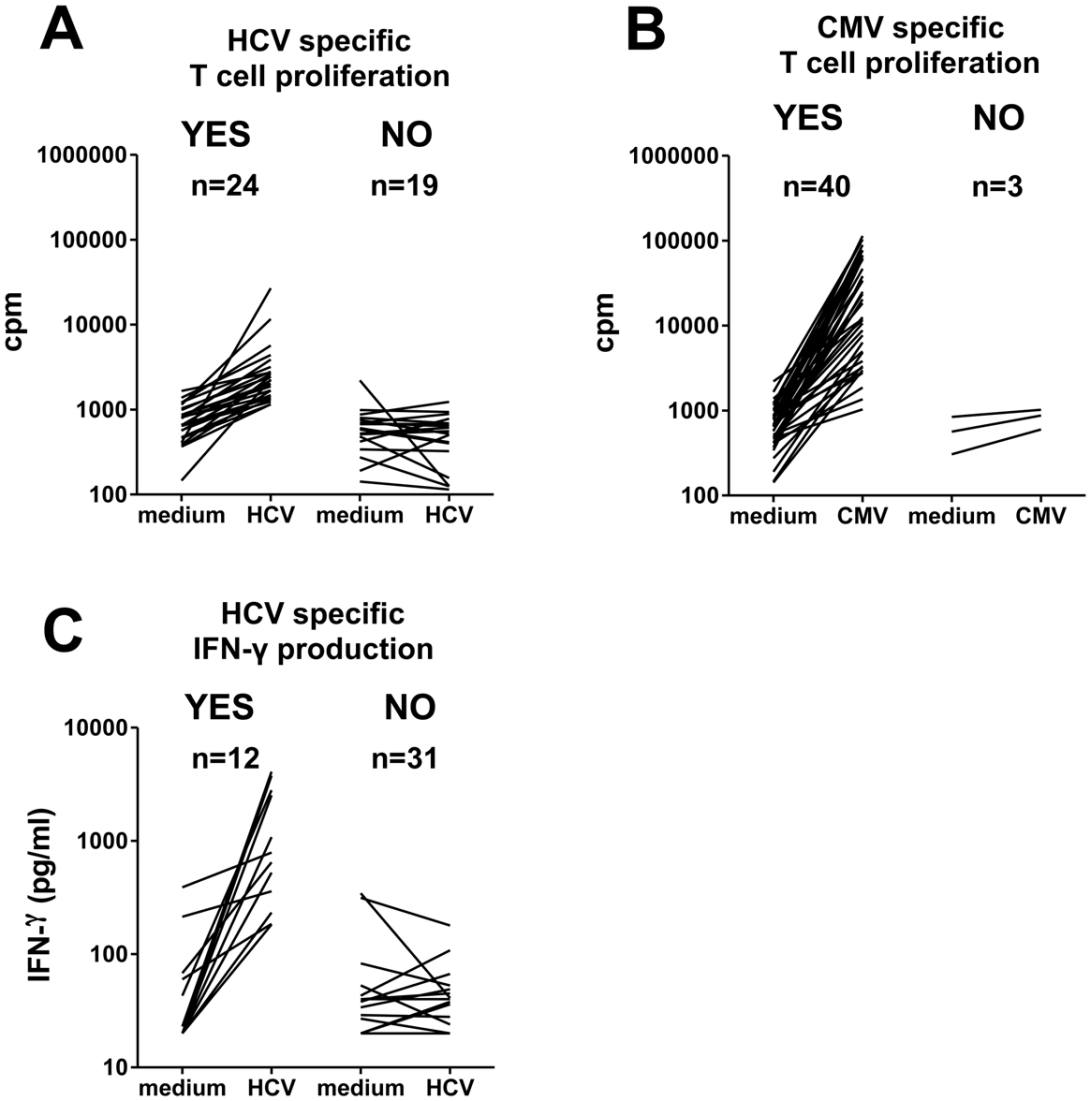
Chronic HCV infected patients show dysfunctional HCV-specific T-cell responses. Forty-three chronic HCV-infected patients were investigated (21 therapy-naive and 22 PegIFN-α/ribavirin experienced). T-cell proliferation (cpm) to HCV (**A**) or CMV (**B**) were determined at day 5 upon stimulation of PBMC, and IFN-γ production (**C**) at day 3. The sensitivity of the IFN-γ ELISA was 20 pg/mL, and therefore data of 17 patients are shown as one single horizontal line in the graph. For all graphs, patients showing significant responses are depicted to the left (YES) and those without are shown to the right (NO).

The strength of T-cell responses to HCV antigens was evaluated in 43 chronic HCV patients. In line with previous reports (reviewed in [1], [2]) upon stimulation of PBMC with a cocktail of HCV peptides, proliferative responses were weak in 24 out of 43 chronic HCV patients, and undetectable in the remaining 19 (Figure 1A). Despite the fact that HCV-specific T-cell responses were weak or undetectable, up to 100 fold stronger CMV-specific T-cell proliferation was detected in PBMC of 40 out of 43 chronic HCV patients (Figure 1B). HCV-specific IFN-γ production was also low to undetectable in most patients, as only 12 out of 43 patients showed substantial IFN-γ production (Figure 1C). Although we mainly analyzed overall T cell responses in our study, in those chronic HCV patients with exceptionally strong HCV-specific responses, HCV peptides were able to elicit simultaneous proliferation and IFN-γ production by both CD4^+^ and CD8^+^ T cells (Figure S2). PBMC from healthy individuals did not respond to the HCV peptide pool (data not shown).

### Multiple Regulatory Mechanisms Suppress HCV-specific T-cell Proliferation in Chronic HCV Patients

**Figure 2:**
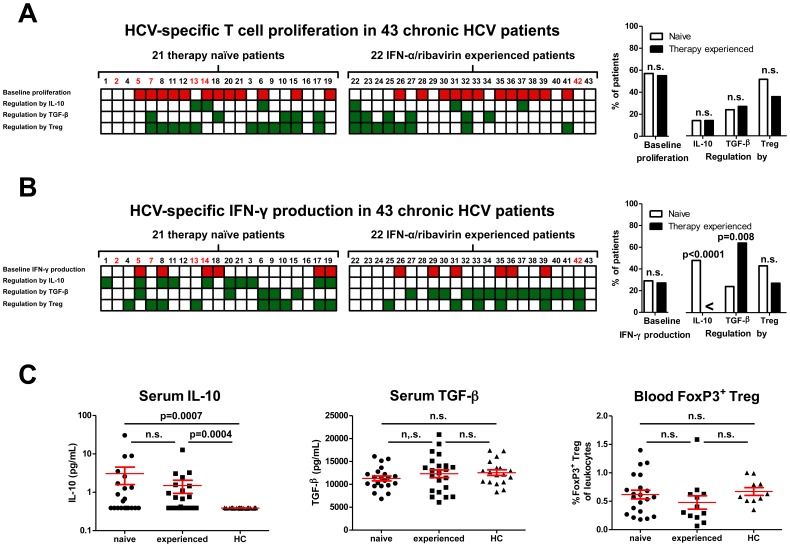
Multiple regulatory mechanisms control HCV-specific T-cell reactivity in PBMC from chronic HCV infected patients. (**A**) Individual data of 43 patients on regulation of HCV-specific T- cell proliferation and (**B**) IFN-γ production are shown (number 1–21 are therapy-naive; 22–43 PegIFN-α/ribavirin experienced patients). Patient numbers are identical to the numbers in Table 1, and numbers of non-genotype 1 patients are depicted in red. Red squares depict HCV-specific T-cell proliferation or IFN-γ production without *in vitro* blockade of any regulatory pathway (baseline response). Green squares reflect patients with a significant increase of HCV-specific responses after IL-10R or TGF-β neutralization, or depletion of Treg. White squares reflect the absence of a baseline response or regulation. The experiments were performed similar as in Figure 1A. Histograms to the right hand side show percentages of patients with HCV-specific responses at baseline, or significant regulation of these responses by respectively IL-10, TGF-β or Treg. The quantitative data of T cell proliferation and IFN-γ production is presented as cpm and IFN-γ levels in Figure S3 to allow evaluation of the strength of the individual responses. (**C**) Serum IL-10 and TGF-β levels and blood CD4+FoxP3+ Treg as a proportion of leukocytes are presented in naïve and treatment experienced patients and healthy controls.

Next, we evaluated the regulation by IL-10, TGF-β and Treg on the dysfunctional HCV-specific T-cell reactivity in the 43 patients. For this purpose, we first determined HCV-specific T-cell proliferation with or without *in vitro* blockade of the IL-10R or TGF-β using neutralizing antibodies, or depletion of CD4^+^CD25^hi^Treg. In this cross-sectional study, suppression by IL-10, TGF-β or Treg of HCV-specific T-cell proliferation was operational in respectively 6, 11 and 19 out of 43 patients (Figure 2A). We are confident that we analyzed the impact of depletion of CD4^+^CD25^hi^ Treg and not the depletion of CD8^+^CD25^hi^FoxP3^+^ Treg, as CD8^+^CD25^hi^ Treg were virtually absent in peripheral blood. The regulation of HCV-specific proliferation was highly heterogeneous in that each of 8 possible combinations of regulatory mechanisms occurred, albeit some at low frequency. Furthermore, no dominant pattern could be identified, and regulation of HCV-specific proliferation and IFN-γ production were often by different mechanisms. Interestingly, HCV-specific T-cell proliferation was not affected by a previous nonresponse to PegIFN-α/ribavirin therapy, as the frequency (Figure 2A) and strength (Figure S3) of regulation by IL-10, TGF-β and Treg was comparable to therapy-naive chronic HCV patients.

### Regulation of HCV-specific IFN-γ Production Differs between PegIFN-α/ribavirin Experienced and Therapy-naive Chronic HCV Patients

Next, we studied whether HCV-specific IFN-γ production in chronic HCV patients was suppressed by multiple regulatory mechanisms similar as the heterogeneous regulation of HCV-specific T-cell proliferation. As shown in Figure 2B, blocking the suppressive effects of IL-10, TGF-β, or Treg enhanced HCV-specific IFN-γ production, albeit that a different pattern of regulation was observed between therapy-naive and PegIFN-α/ribavirin experienced chronic HCV patients. For therapy-naive chronic HCV patients, similar as for T-cell proliferation, regulation of HCV-specific IFN-γ production was highly heterogeneous.

In sharp contrast, in PegIFN-α/ribavirin experienced chronic HCV patients IL-10 mediated regulation of IFN-γ production was absent (Figure 2B). Moreover, different from therapy-naive patients, TGF-β suppressed HCV-specific IFN-γ production in the majority of therapy-experienced chronic HCV patients (Figure 2B; respectively 5 out of 21 and 14 out of 22) with strong increments in IFN-γ levels detected after blocking TGF-β (Figure S3B). Regulation by Treg was also observed in PegIFN-α/ribavirin experienced patients, however at similar rates and with comparable strength as for therapy-naive patients.

Levels of circulating TGF-β and Treg frequencies in blood were similar for therapy-naive patients, PegIFN-α/ribavirin experienced patients and healthy controls (Figure 2C) and did not correlate with HCV-specific T-cell reactivity (data not shown). Also, no correlation was observed between the levels of circulating TGF-β and Treg frequencies in blood and the effects of TGF-β neutralization or Treg depletion. In contrast to healthy individuals, enhanced serum IL-10 was measured in some, but not all, HCV-infected patients (Figure 2C). However, the presence of serum IL-10 did not correlate with clinical parameters such as HCV-RNA or ALT levels, or with the presence of IL-10 dependent regulation of HCV-specific T-cell proliferation or IFN-γ production (data not shown).

### IL-10 Mediated Suppression of HCV-specific IFN-γ Production Affects the Level of HCV Replication in Therapy-naive Patients

**Figure 3:**
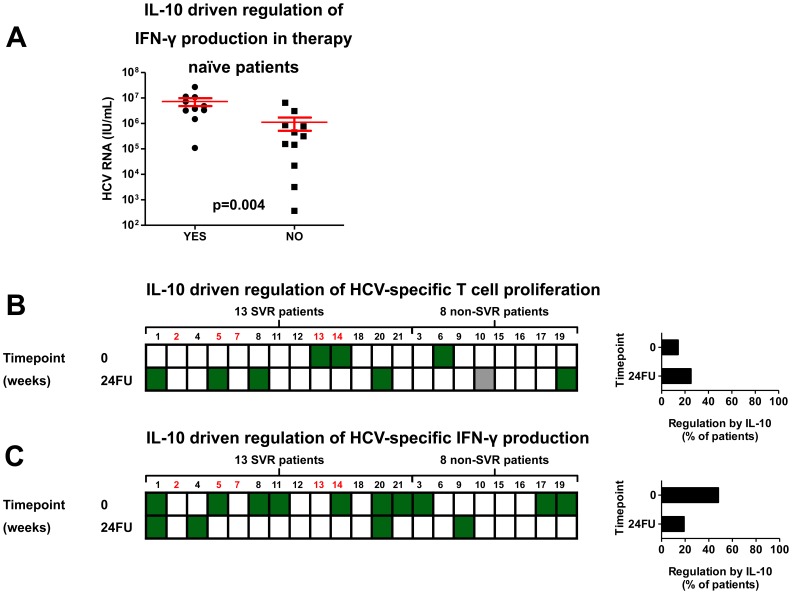
Regulation of HCV-specific immunity by IL-10 is associated with higher HCV-RNA levels in therapy-naive patients, however not linked to success of PegIFN-α/ribavirin therapy. (**A**) HCV-RNA levels of 21 therapy-naive chronic HCV patients with or without regulation by IL-10 of HCV-specific IFN-γ production. (**B**) Individual data of 21 previously therapy-naive chronic HCV patients before and 24 weeks after ending therapy are shown. Patients 1–13 showed an SVR, patients 14–21 did not achieve an SVR (non-SVR). (**A**) Green squares reflect patients with a significant increase in either HCV-specific proliferation or (**B**) IFN-γ production after neutralization of the IL-10R. White squares reflect the absence of regulation by IL-10. Grey squares reflect missing data. Histograms to the right side show percentages of patients with significant IL-10 driven regulation of HCV-specific responses at the indicated timepoints.

To identify mechanisms explaining clinical differences between patients, we questioned whether regulation of HCV-specific T-cell reactivity was related to disease parameters. As shown in Figure 3A, HCV-RNA levels in therapy-naive patients were significantly higher in case of active IL-10 mediated suppression of HCV-specific IFN-γ production, as opposed to the lower HCV-RNA levels observed in therapy-naive patients without IL-10 driven regulation of HCV-specific IFN-γ production (p<0.004). Other significant associations between regulation by IL-10, TGF-β, or Treg and disease parameters such as time-since-infection, ALT levels, and liver fibrosis grade were not found, neither for the whole group of 43 chronic HCV patients, nor for therapy-naive or PegIFN-α/ribavirin experienced patients separately (data not shown). To increase the power of our analysis, we included a group of 23 additional chronic HCV patients (Table S1 for patient characteristics), and retested for correlation with disease parameters. Re-analysis of this extended data-set (n = 66) revealed similar results (data not shown).

### Irrespective of Viral Outcome, Regulation of HCV-specific IFN-γ Production by TGF-β Increases During and up to 24 Weeks After PegIFN-α/ribavirin Therapy

There is an ongoing debate whether PegIFN-α/ribavirin therapy restores the dysfunctional HCV-specific T-cell response due to removal of antigenic pressure on immune cells [23]. If this is the case, the dynamics in activity of regulatory pathways may explain this restoration. To examine this in our cohort, we prospectively examined the type and degree of regulation of HCV-specific T-cell responses at various timepoints during, and up to 24 weeks after therapy in 21 therapy-naive patients receiving PegIFN-α/ribavirin therapy for the first time. When pretreatment and 24 weeks after therapy were compared, regulation of HCV-specific T-cell proliferation by IL-10 was never observed at both timepoints and only present in a small proportion of patients (Figure 3B). Also during therapy, only a minority of patients showed regulation of HCV-specific T-cell proliferation by IL-10. At the same time, IL-10 driven suppression of HCV-specific IFN-γ production remained present during and shortly after therapy, but decreased 24 weeks after therapy, both in patients achieving a sustained viral response (SVR) and in non-SVR patients (Figure 3C and Figure S4). Regulation of HCV-specific T-cell reactivity by IL-10 was not related to the outcome of therapy, as regulation of HCV-specific T-cell proliferation and IFN-γ production was equally distributed among the 13 SVR patients and 8 non-SVR patients.

**Figure 4:**
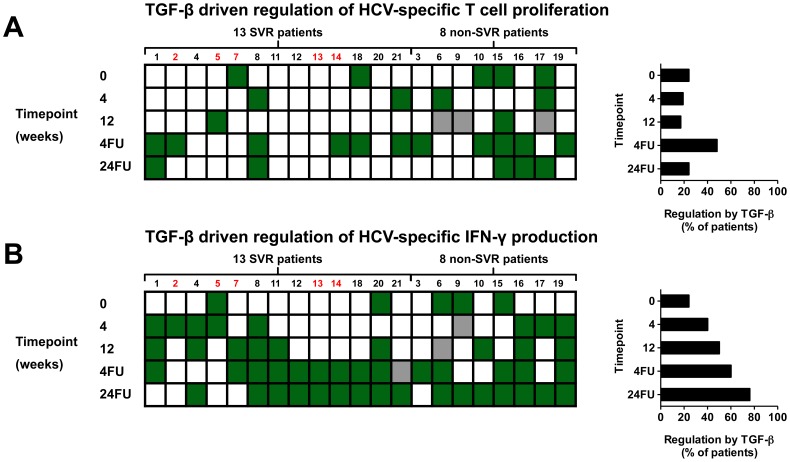
Irrespective of viral outcome, regulation of HCV-specific IFN-γ production by TGF-β increases during and up to 24 weeks after PegIFN-α/ribavirin therapy. Individual data of previously therapy-naive chronic HCV patients before, during and after therapy are shown. Patients 1–13 showed an SVR, patients 14–21 did not achieve an SVR (non-SVR). (**A**) Green squares reflect patients with a significant increase in either HCV-specific proliferation or (**B**) IFN-γ production after neutralization of TGF-β. White squares reflect the absence of regulation by TGF-β. Grey squares reflect missing data. Histograms to the right side show percentages of patients with significant TGF-β driven regulation of HCV-specific responses at the indicated timepoints.

Suppression of HCV-specific T-cell proliferation by TGF-β was infrequent during and 24 weeks after therapy as it was before therapy. Only at 4 weeks after end of therapy, a transient increase in regulation of HCV-specific T-cell proliferation by TGF-β was observed (Figure 4A). In contrast, although serum TGF-β levels remained unchanged (data not shown), TGF-β driven regulation of HCV-specific IFN-γ production increased gradually during therapy and continued to increase up to 24 weeks after therapy, when 16 out of 21 patients showed regulation by TGF-β (Figure 4B). Twenty-four weeks after therapy, regulation was not only most frequent, but also strong as TGF-β neutralization resulted in a mean increase of HCV-specific IFN-γ production of 3111 pg/mL.

## Discussion

This study establishes that the regulation by IL-10, TGF-β and Treg of dysfunctional HCV-specific immunity in chronic HCV patients is highly heterogeneous. However, two clear differences between PegIFN-α/ribavirin experienced patients and therapy-naive patients could be distinguished. In therapy-experienced chronic HCV patients, regulation of HCV-specific IFN-γ production by TGF-β was dominant, while regulation by IL-10 was absent. In contrast, in about half of therapy-naive chronic HCV patients, especially those with a high viral load, IL-10 was involved in the regulation of HCV-specific IFN-γ production. Upon treatment with PegIFN-α/ribavirin of previously therapy-naive patients, regulation by TGF-β of HCV-specific IFN-γ production gradually increased over time and reached a maximum at the end of follow-up at 24 weeks after therapy.

The present study clearly shows that Treg, TGF-β and IL-10 all contribute to the suppression of HCV-specific T-cell proliferation in chronic HCV patients. Our data suggest that regulation of HCV-specific T cells by IL-10, TGF-β or Treg was specific for HCV, as the pattern of regulation of HCV-specific T cells differed from regulation of CMV-specific T cells (Figure S5). Although our findings on the regulation of virus-specific T cell proliferation suggest that IL-10 is more important to control CMV responses as compared to HCV responses, one should be aware that the nature of the virus antigen differs (peptide pool versus CMV lysate). Importantly, also in those patients in whom HCV-specific T-cell proliferation or IFN-γ production was not observed upon stimulation with HCV peptides alone, these responses could be revived by blocking one of the inhibitory mechanisms studied. However, we now show that the importance of the specific pathways to inhibit HCV-specific immunity differs between patients and no dominant regulatory mechanism can be identified. This strong heterogeneity of regulation of HCV-specific immunity has not been emphasized before. In contrast, previous reports suggested exclusive suppression of HCV-specific immune responses by Treg, TGF-β or IL-10 [10], [11], [12], [13], [14], [16], [18] and to our knowledge only three previous reports showed involvement of both TGF-β and IL-10 in the suppression of HCV-specific immunity [17], [19]. In addition, the recent paper by Raziorrouh and colleagues suggests that inhibition of IL-10 and TGF-β simultaneously is the most promising condition to restore HCV-specific T-cell responses [5]. Our present study is complementary and is in agreement with these findings when evaluating the mechanisms of negative regulation at baseline, although we did not block IL-10 and TGF-β simultaneously. Importantly, in our study, we further emphasize the heterogeneous nature of the regulatory pathways, and also the modulation of the regulatory mechanisms during the course of IFN-α-based antiviral therapy. Differential expression of IL-10 or TGF-β receptors on HCV-specific cells between different patients and between different antigen specificities is a possible mechanism responsible for the heterogeneity of regulation. The heterogeneous character of regulation of HCV-specific T-cell immunity is reminiscent of the expression of exhaustion markers and their functional synergy to control HCV-specific T-cell immunity [26]–[27].

One possible explanation for the heterogeneity of regulation is that patients are at different phases of disease, and vary greatly in level of viral replication and viral sequences, liver inflammation, pathology and other disease parameters. Additionally, in our study cohort consisting of 43 patients, 6 non-HCV genotype-1 patients were included, and we can not exclude that PBMC from these patients may show a distinct cross-reactivity to the peptides resulting in heterogeneity in immune responses. We suggest that time-since-infection, and subsequent differences in phase of disease are important factors explaining the heterogeneous regulation of T-cell responses in chronic and therapy-induced resolved HCV infection. This has been proposed before as a general model for regulation of immunity to chronic infections [4]. In line with this concept, our study shows that HCV-RNA loads were higher in chronic HCV patients showing active IL-10 mediated suppression of HCV-specific IFN-γ production. We cannot explain why individual patients differ with respect to the usage of IL-10 pathways leading to differences in HCV-RNA levels, but genetic polymorphisms in the IL-10 gene may be important. Although we determined the IL-28B, IL-10 and TGF-β gene polymorphisms (see Table 1), our study cohort was too small to draw firm conclusions on the importance of these SNPs in modulating T cell reactivity to HCV. A larger patient cohort and more detailed information on time-since-infection is needed to establish further relations between disease parameters and regulation of HCV-specific immunity.

This is the first prospective study examining PegIFN-α/ribavirin therapy-induced effects on IL-10 and TGF-β mediated suppression of HCV-specific immune responses. We observed that upon antiviral treatment IL-10 mediated regulation was diminished and TGF-β mediated regulation strongly enhanced. Irrespective of response to therapy, IL-10 driven regulation of HCV-specific IFN-γ production was decreased 24 weeks after therapy, albeit that a small group of patients still showed IL-10 regulation. Possibly, a longer follow-up is needed to loose IL-10 driven regulation of HCV-specific IFN-γ production, as our cohort of 22 chronic HCV infected patients with a previous nonresponse to therapy showed an absence of IL-10 driven regulation of HCV-specific IFN-γ production (Figure 2b). As mentioned above, IL-10 driven regulation may allow higher levels of HCV replication in therapy-naive chronic HCV patients. It seems that exposure to PegIFNα/ribavirin therapy diminishes the importance of this IL-10 mediated suppression of anti-HCV immunity irrespective of virologic response to therapy.

Our prospective data showed that when therapy-naive patients were treated with PegIFN-α/ribavirin, regulation by TGF-β of HCV-specific IFN-γ production gradually increased over time, and reached a maximum at the end of follow-up at 24 weeks after therapy. Thus, exposure to PegIFN-α/ribavirin increases the frequency of TGF-β driven regulation of HCV-specific IFN-γ production. This may be a direct effect of PegIFN-α/ribavirin therapy and not secondary to a decrease in HCV-RNA load, since regulation by TGF-β increases gradually in both SVR and non-SVR patients. Our finding that TGF-β driven regulation of HCV-specific IFN-γ production increases induced by PegIFN-α/ribavirin, is supported by our cross-sectional data on previous nonresponders to therapy, who still show frequent regulation by TGF-β. Also when these previous nonresponders are retreated, regulation by TGF-β remains dominant and regulation by IL-10 remains rare (data not shown). At present there is only limited information available on the interplay between IFN-α and TGF-β responses. However, in studies using rats, it was shown that IFN-α-2b can increase TGF-β1 production [28] and that positive cross talk exists between IFN-α and TGF-β1 signaling [29]. These observations may at least in part explain our findings since they are in line with the more frequent regulation of T cell responses by TGF-β we describe in our manuscript. The importance of TGF-β in the nonresponse to IFN-α-based treatment of chronic HCV patients has been reported before. It has been demonstrated, using gene expression analysis, that TGF-β pathways – similar to the interferon stimulated gene pathways– are already upregulated at baseline in non-responder patients [30].

For SVR patients, TGF-β mediated suppression of HCV-specific IFN-γ production by memory T-cells was unexpected, since the activity of TGF-β in HCV is often linked to liver fibrogenesis [31] and SVR is associated with a reduction in fibrosis [32]. Importantly, total TGF-β concentrations in serum were similar before and after successful HCV eradication (data not shown), in contrast to older studies reporting reduced serum TGF-β levels in SVR patients [33], [34]. Our study indicates a role for TGF-β as a regulatory cytokine suppressing the pro-inflammatory responses after PegIFN-α/ribavirin therapy, irrespective of viral response, rather than a cytokine promoting liver fibrogenesis. The *in vivo* situation where chronic HCV patients are treated for prolonged periods of time with PegIFN-α/ribavirin leads to reduction of HCV-RNA levels and ideally to eradication of the virus and reduction of inflammatory processes. However, since the receptors for IFN-α are expressed on most leukocyte populations as well as non-leukocytes, the effects are broad and the antiviral mechanisms of action of IFN-α are numerous. Since the *in vivo* situation is extremely complex, *in vitro* experiments to corroborate the effect of PegIFN-α/ribavirin on TGF-β production will always be an oversimplification of the situation in patients. Also, it is worth mentioning that in our experimental setup, the source of TGF-β and IL-10 is unknown and not necessarily derived from T cells.

Treg mediated regulation of HCV-specific proliferation gradually decreased during and after PegIFN-α/ribavirin therapy in both SVR and non-SVR patients, while regulation by Treg of HCV-specific IFN-γ production remained relatively stable (Figure S6). These data are in line with the only previous study that prospectively examined regulation by Treg during PegIFN-α/ribavirin therapy that did not find a role for peripheral blood Treg in the response to treatment [35]. As the actual depletion of CD4^+^CD25^+^FoxP3^+^ Treg using CD25 magnetic beads was at least 80%, a small fraction of Treg remained present, Therefore, our study may underestimate the importance of Treg in regulating HCV-specific responses to a certain extent. In future studies, depletion of Treg by flowcytometric sorting and inclusion of CD127 can improve this percentage. Moreover, in contrast to previous studies, we did not observe increased numbers of Treg in HCV-infected patients compared to healthy individuals, which may be due to the inclusion of the FoxP3 marker to define Treg in contrast to other studies [10], [11], [12], [13]. In contrast to blood, we recently showed that intrahepatic CD4^+^CD25^+^FoxP3^+^ Treg increased during therapy, and their numbers remained elevated for more than 6 months after therapy-induced viral clearance in the majority of patients [9].

Additional research on the complex process of heterogeneous coregulation is required to understand the dysfunctional HCV-specific immunity in chronic HCV patients. In this, the use of CD4 tetramers, which have recently been described [36], would be instrumental to obtain more detailed mechanistic data, for instance on the regulation of IL-10 and TGF-β receptor expression on HCV-specific T cells. Further evaluation of multiple regulatory mechanisms may explain variability in immunopathology between chronic HCV patients, and optimization of immunotherapy should take into account the substantial differences between patients with respect to inhibitory processes preventing protective immunity against HCV.

## Supporting Information

Figure S1 **Flowchart of patients included in the study.** 43 Chronic HCV infected patients were included: therapy-naive (n = 21) and PegIFN-α/ribavirin experienced (n = 22). A cross-sectional immunological study was carried out on these 43 patients. Therapy-naive chronic HCV patients received standard PegIFN-α/ribavirin therapy. Thirteen patients achieved an SVR, as they remained HCV-RNA negative 6 months after end of therapy. Eight patients showed a nonresponse to therapy, and did not become HCV-RNA negative. A longitudinal immunological study was carried out in which the 21 therapy-naive patients were followed up during and up to 24 weeks after therapy.
(PDF)

Figure S2 **HCV peptides can elicit simultaneous proliferation and IFN-γ production by both CD4^+^ and CD8^+^ T cells.** Frequencies of IFN-γ producing HCV-specific CD4^+^ or CD8^+^ T cells were determined using flowcytometry. PBMC labelled with CFSE (0.25 µM; Invitrogen) were cultured in 24-well flat bottom plates (10^6^ cells in 1 mL) in the presence or absence of the HCV peptide pool. At day 6, cells were restimulated in 24-well plates coated with anti-CD3 (5 µg/mL; OKT-3, Janssen-Cilag) for 2 hours and an additional 3 hours with Brefeldin-A (10 µg/mL; Sigma-Aldrich). Cells were fixed (2% formaldehyde, 20 minutes), permeabilized (0.5% saponin) and labelled with CD4-APC-H7 (SK3; BD, San Jose, USA), CD8-PerCP (RPA-T8; eBioscience) and IFN-γ-PE-Cy7 (4S.B3; BD).
(PDF)

Figure S3 **Multiple regulatory mechanisms control HCV-specific T-cell reactivity in PBMC from chronic HCV infected patients.** Quantitative data on **(A)** regulation of HCV-specific T-cell proliferation (cpm) and **(B)** IFN-γ production (pg/mL) are shown for 43 chronic HCV patients (21 therapy-naive and 22 PegIFN-α/ribavirin therapy experienced). Graphs to the left, middle and right, respectively, show the effects of neutralization of the IL-10R or TGF-β, or depletion of Treg. The experiments were performed similar as in Figure 1A and 2. For all graphs, the number of patients with and without regulation is given (YES, n = number and NO, n = number, respectively). Data before and after neutralization of the IL-10R or TGF-β, or depletion of Treg were compared using Student’s t-test for paired data or the Wilcoxon matched pairs test, where appropriate.
(PDF)

Figure S4 **Frequency of regulation by IL-10 of HCV-specific T-cell proliferation is stable during PegIFN-α/ribavirin therapy and regulation of HCV-specific IFN-γ production by IL-10 is decreased at 24 weeks after PegIFN-α/ribavirin therapy.** Individual patient data before (T = 0), week 4 or 12 during (T = 4 and T = 12, respectively) and 4 or 24 weeks after PegIFN-α/ribavirin therapy (T = 4FU and T = 24FU, respectively) are shown for 21 previously therapy-naive chronic HCV patients. Patients 1 to 13 showed a sustained viral response, patients 14 to 21 a viral nonresponse. **(A)** Green squares reflect patients with a significant increase in either HCV-specific proliferation or **(B)** IFN-γ production after neutralization of IL-10R. White squares reflect the absence of regulation by IL-10. Grey squares reflect missing data. Histograms to the right side show percentages of patients with significant IL-10 driven regulation of HCV-specific responses at the indicated timepoints.
(PDF)

Figure S5 **Regulation of HCV-specific T cells by IL-10, TGF-β or Treg was specific for HCV, as the pattern of regulation differed completely from regulation of CMV-specific T cells. (A)** Individual data of 43 patients on regulation of HCV-specific T- cell proliferation and **(B)** regulation of CMV-specific T- cell proliferation are shown (number 1–21 are therapy-naive; 22–43 PegIFN-α/ribavirin experienced patients). Patient numbers are identical to the numbers in the Table 1 and numbers of non-genotype 1 patients are depicted in red. Red squares depict CMV or HCV-specific T-cell proliferation without *in vitro* blockade of any regulatory pathway (baseline response). Green squares reflect patients with a significant increase of antigen-specific responses after IL-10R or TGF-β neutralization, or depletion of Treg. White squares reflect the absence of a baseline response or regulation. Grey squares reflect the absence of data. The experiments were performed similar as in Figure 1A.
(PDF)

Figure S6 **Irrespective of viral outcome, regulation of HCV-specific T-cell proliferation by Treg decreases during and up to 24 weeks after PegIFN-α/ribavirin therapy.** Individual patient data before (T = 0), week 4 or 12 during (T = 4 and T = 12, respectively) and 4 or 24 weeks after PegIFN-α/ribavirin therapy (T = 4FU and T = 24FU, respectively) are shown for 21 previously therapy-naive chronic HCV patients. Patients 1 to 13 showed an SVR, patients 14 to 21 a viral nonresponse. **(A)** Green squares reflect patients with a significant increase in either HCV-specific proliferation or **(B)** IFN-γ production after depletion of Treg. White squares reflect the absence of regulation by Treg. Grey squares reflect missing data. Histograms to the right show percentages of patients with significant Treg driven regulation of HCV-specific responses at the indicated timepoints.
(PDF)

Table S1 **Patient characteristics additional patients (n  = 23).**
(PDF)
