# Supplementary figures and images for: Negative Regulation of Hepatitis C Virus Specific Immunity Is Highly Heterogeneous and Modulated by Pegylated Interferon-Alpha/Ribavirin Therapy
Source: PLoS One. 2012 Nov 8;7(11):e49389. doi: 10.1371/journal.pone.0049389 (PMC3493527; doi:10.1371/journal.pone.0049389)

# Supplementary Figure 1

Flowchart of patients included in the study.

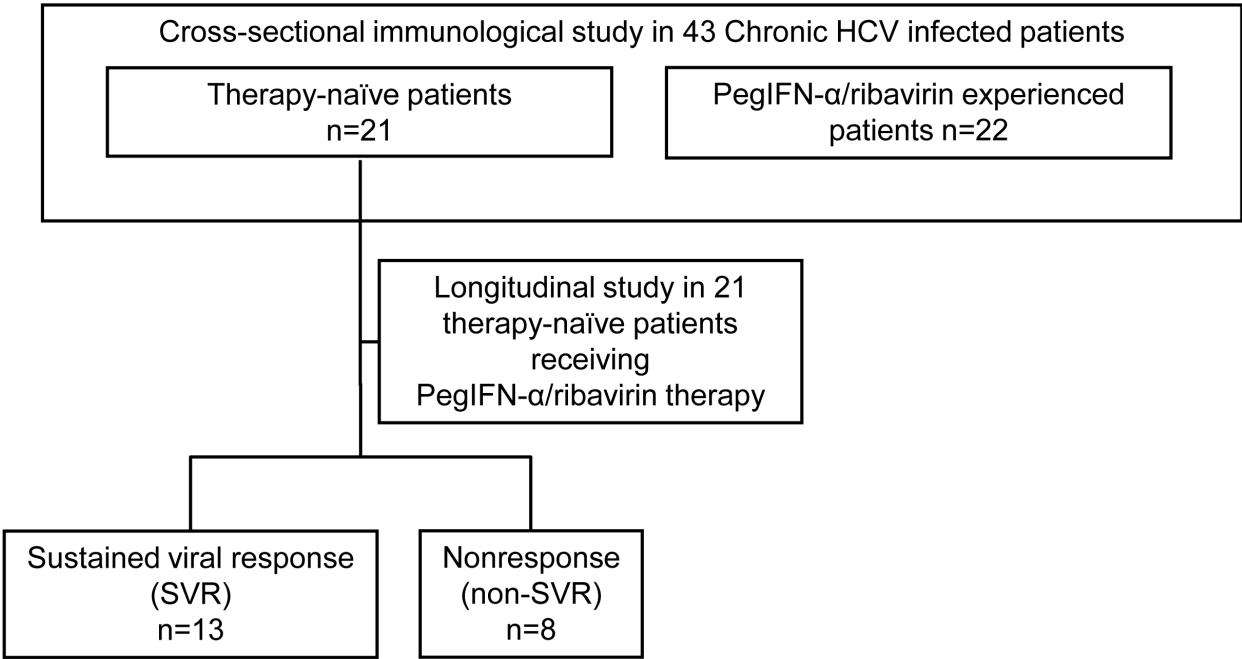

Supplement: Figure S1 — Flowchart of patients included in the study. 43 Chronic HCV infected patients were included: therapy-naive (n = 21) and PegIFN-α/ribavirin experienced (n = 22). A cross-sectional immunological study was carried out on these 43 patients. Therapy-naive chronic HCV patients received standard PegIFN-α/ribavirin therapy. Thirteen patients achieved an SVR, as they remained HCV-RNA negative 6 months after end of therapy. Eight patients showed a nonresponse to therapy, and did not become HCV-RNA negative. A longitudinal immunological study was carried out in which the 21 therapy-naive patients were followed up during and up to 24 weeks after therapy. (PDF) [file pone.0049389.s001.pdf]

Supplementary Figure 2

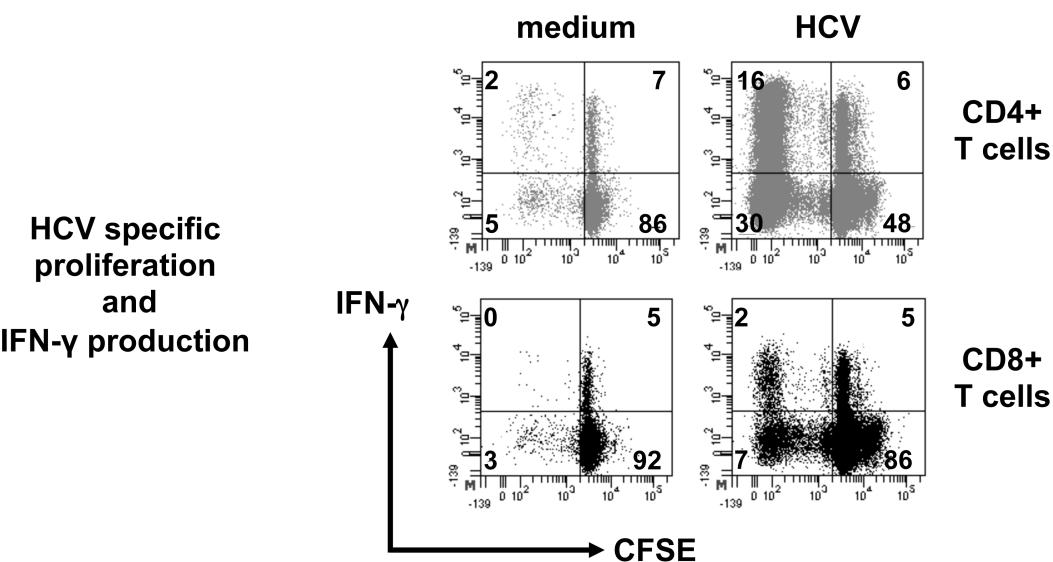

Supplement: Figure S2 — HCV peptides can elicit simultaneous proliferation and IFN-γ production by both CD4+ and CD8+ T cells. Frequencies of IFN-γ producing HCV-specific CD4+ or CD8+ T cells were determined using flowcytometry. PBMC labelled with CFSE (0.25 µM; Invitrogen) were cultured in 24-well flat bottom plates (106 cells in 1 mL) in the presence or absence of the HCV peptide pool. At day 6, cells were restimulated in 24-well plates coated with anti-CD3 (5 µg/mL; OKT-3, Janssen-Cilag) for 2 hours and an additional 3 hours with Brefeldin-A (10 µg/mL; Sigma-Aldrich). Cells were fixed (2% formaldehyde, 20 minutes), permeabilized (0.5% saponin) and labelled with CD4-APC-H7 (SK3; BD, San Jose, USA), CD8-PerCP (RPA-T8; eBioscience) and IFN-γ-PE-Cy7 (4S.B3; BD). (PDF) [file pone.0049389.s002.pdf]

# Supplementary Figure 3

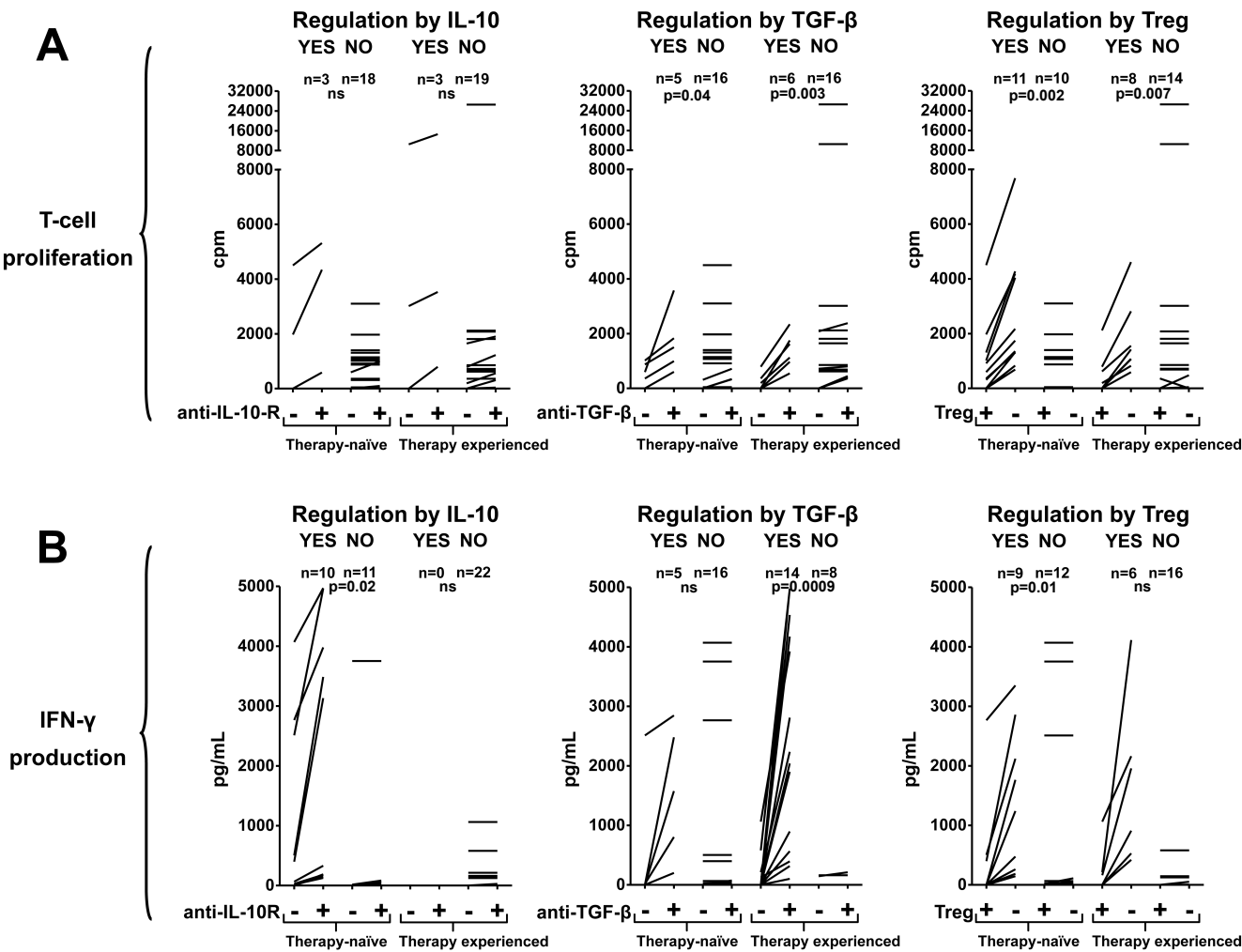

Supplement: Figure S3 — Multiple regulatory mechanisms control HCV-specific T-cell reactivity in PBMC from chronic HCV infected patients. Quantitative data on (A) regulation of HCV-specific T-cell proliferation (cpm) and (B) IFN-γ production (pg/mL) are shown for 43 chronic HCV patients (21 therapy-naive and 22 PegIFN-α/ribavirin therapy experienced). Graphs to the left, middle and right, respectively, show the effects of neutralization of the IL-10R or TGF-β, or depletion of Treg. The experiments were performed similar as in Figure 1A and 2. For all graphs, the number of patients with and without regulation is given (YES, n = number and NO, n = number, respectively). Data before and after neutralization of the IL-10R or TGF-β, or depletion of Treg were compared using Student’s t-test for paired data or the Wilcoxon matched pairs test, where appropriate. (PDF) [file pone.0049389.s003.pdf]

# Supplementary Figure 4

A

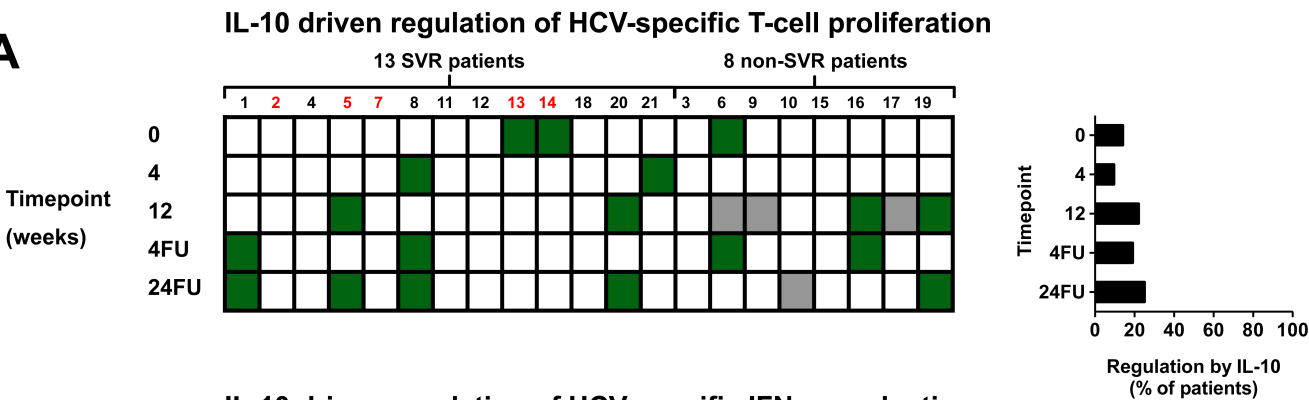

B

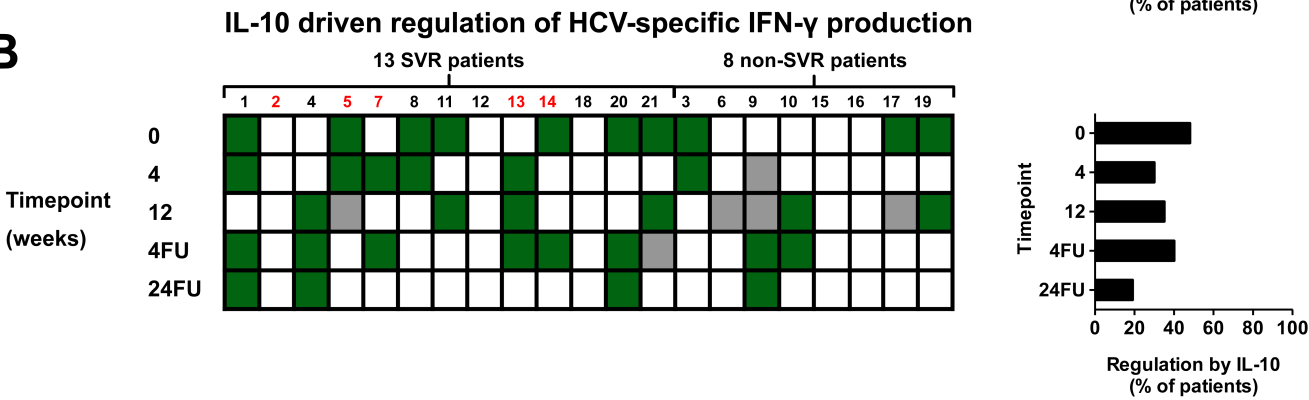

Supplement: Figure S4 — Frequency of regulation by IL-10 of HCV-specific T-cell proliferation is stable during PegIFN-α/ribavirin therapy and regulation of HCV-specific IFN-γ production by IL-10 is decreased at 24 weeks after PegIFN-α/ribavirin therapy. Individual patient data before (T = 0), week 4 or 12 during (T = 4 and T = 12, respectively) and 4 or 24 weeks after PegIFN-α/ribavirin therapy (T = 4FU and T = 24FU, respectively) are shown for 21 previously therapy-naive chronic HCV patients. Patients 1 to 13 showed a sustained viral response, patients 14 to 21 a viral nonresponse. (A) Green squares reflect patients with a significant increase in either HCV-specific proliferation or (B) IFN-γ production after neutralization of IL-10R. White squares reflect the absence of regulation by IL-10. Grey squares reflect missing data. Histograms to the right side show percentages of patients with significant IL-10 driven regulation of HCV-specific responses at the indicated timepoints. (PDF) [file pone.0049389.s004.pdf]

# Supplementary Figure 6

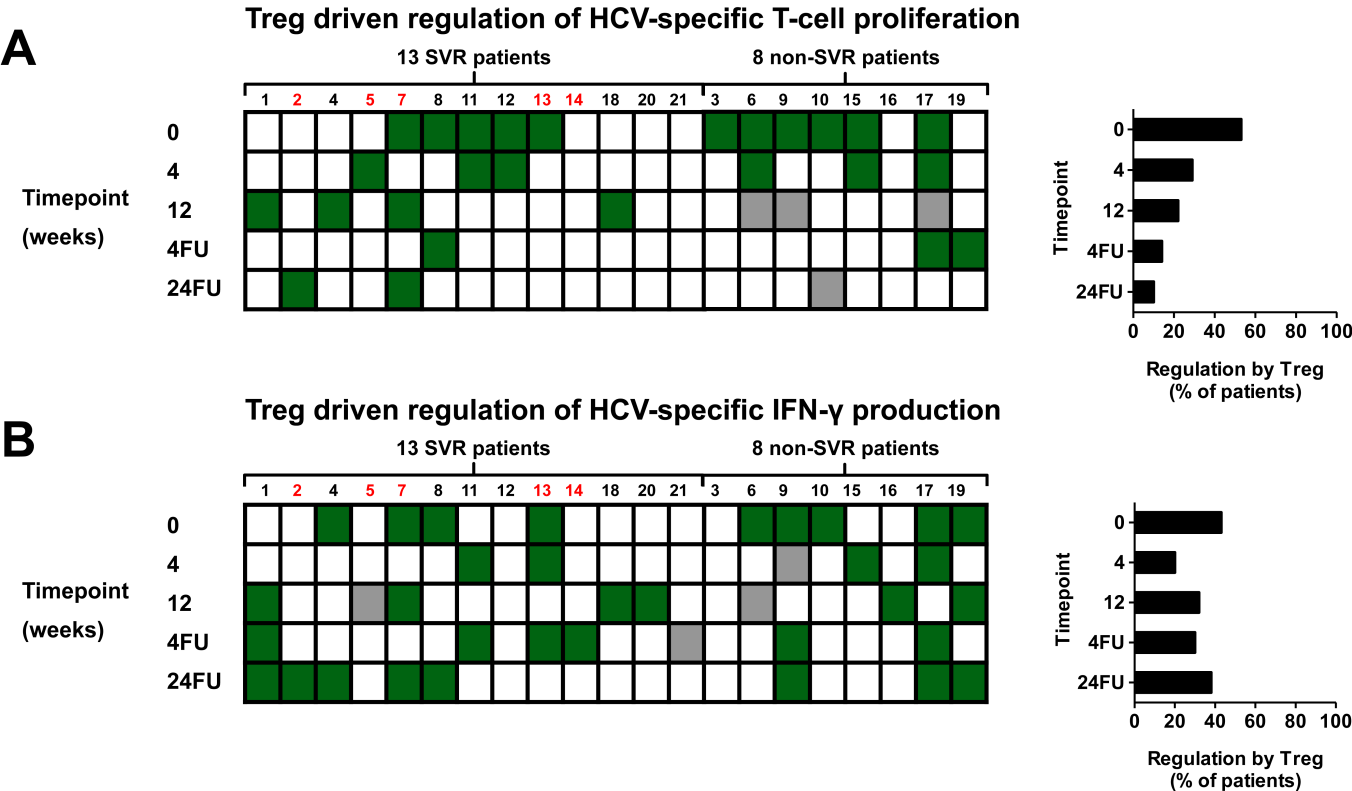

Supplement: Figure S6 — Irrespective of viral outcome, regulation of HCV-specific T-cell proliferation by Treg decreases during and up to 24 weeks after PegIFN-α/ribavirin therapy. Individual patient data before (T = 0), week 4 or 12 during (T = 4 and T = 12, respectively) and 4 or 24 weeks after PegIFN-α/ribavirin therapy (T = 4FU and T = 24FU, respectively) are shown for 21 previously therapy-naive chronic HCV patients. Patients 1 to 13 showed an SVR, patients 14 to 21 a viral nonresponse. (A) Green squares reflect patients with a significant increase in either HCV-specific proliferation or (B) IFN-γ production after depletion of Treg. White squares reflect the absence of regulation by Treg. Grey squares reflect missing data. Histograms to the right show percentages of patients with significant Treg driven regulation of HCV-specific responses at the indicated timepoints. (PDF) [file pone.0049389.s006.pdf]
