# Supplementary material for: Negative Regulation of Hepatitis C Virus Specific Immunity Is Highly Heterogeneous and Modulated by Pegylated Interferon-Alpha/Ribavirin Therapy
Source: PLoS One. 2012 Nov 8;7(11):e49389. doi: 10.1371/journal.pone.0049389 (PMC3493527; doi:10.1371/journal.pone.0049389)

# Supplementary Figure 5

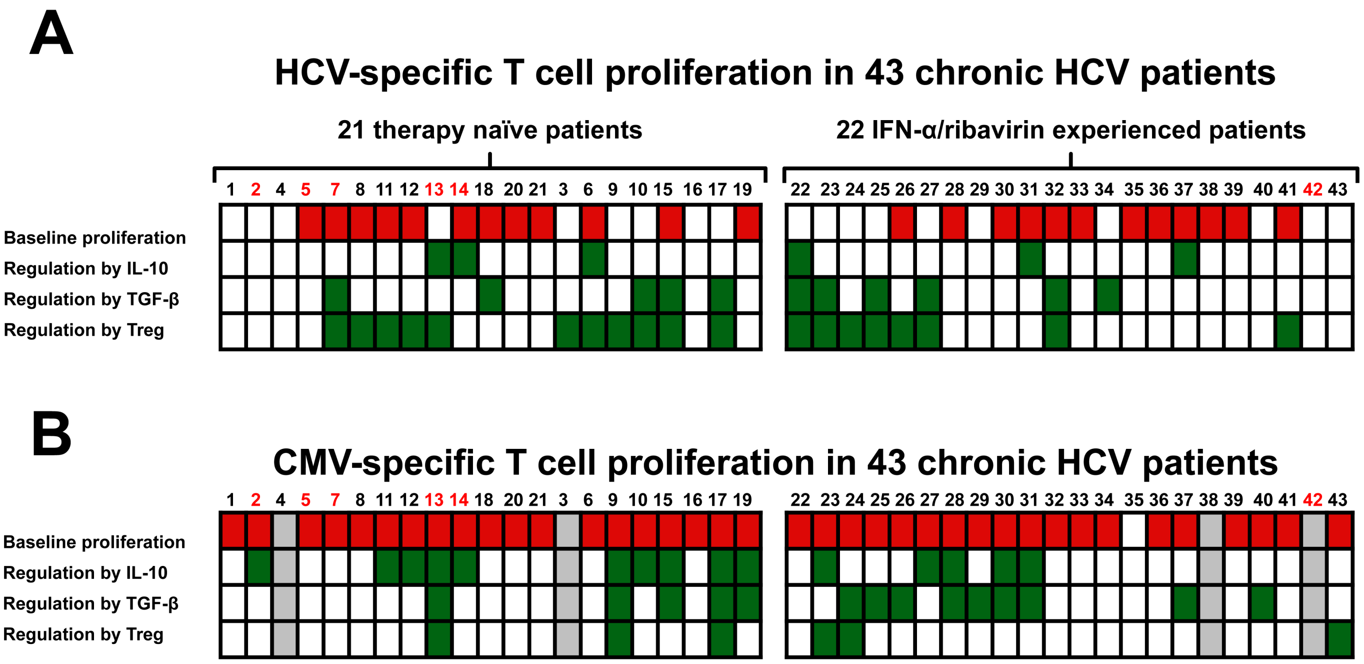

Supplement: Figure S5 — Regulation of HCV-specific T cells by IL-10, TGF-β or Treg was specific for HCV, as the pattern of regulation differed completely from regulation of CMV-specific T cells. (A) Individual data of 43 patients on regulation of HCV-specific T- cell proliferation and (B) regulation of CMV-specific T- cell proliferation are shown (number 1–21 are therapy-naive; 22–43 PegIFN-α/ribavirin experienced patients). Patient numbers are identical to the numbers in the Table 1 and numbers of non-genotype 1 patients are depicted in red. Red squares depict CMV or HCV-specific T-cell proliferation without in vitro blockade of any regulatory pathway (baseline response). Green squares reflect patients with a significant increase of antigen-specific responses after IL-10R or TGF-β neutralization, or depletion of Treg. White squares reflect the absence of a baseline response or regulation. Grey squares reflect the absence of data. The experiments were performed similar as in Figure 1A. (PDF) [file pone.0049389.s005.pdf]
