# Supplementary material for: Negative Regulation of Hepatitis C Virus Specific Immunity Is Highly Heterogeneous and Modulated by Pegylated Interferon-Alpha/Ribavirin Therapy
Source: PLoS One. 2012 Nov 8;7(11):e49389. doi: 10.1371/journal.pone.0049389 (PMC3493527; doi:10.1371/journal.pone.0049389)
Supplement: Table S1 — Patient characteristics additional patients (n = 23). (PDF) [file pone.0049389.s007.pdf]

Supplementary Table 1

Patient characteristics additional patients (n = 23):

Study number	Sex (M/F)	Age (years)	Liver fibrosis (Metavir)	Genotype	HCV RNA (IU/mL)	ALT (U/L)
X1	M	42	n.d.	3	5.19E+06	355
X2	M	46	3	2	1.56E+07	47
X3	M	49	n.d.	1	2,88E+05	20
X4	M	44	n.d.	1	Pos	71
X5	M	73	4	2	1.75E+07	251
X6	M	44	1	1	8,74E+06	48
X7	F	23	2	4	1.53E+06	73
X8	F	62	2	4	1.57E+07	110
X9	M	19	1	1	5.91E+06	42
X10	F	57	4	1	1.61E+06	62
X11	M	48	3	2	Pos	13
X12	M	43	n.d.	2	Pos	51
X13	F	62	1	1	1.28E+06	55
X14	F	68	4	1	Pos	78
X15	F	62	n.d.	1	Pos	49
X16	M	59	4	4	1.70E+04	64
X17	M	56	4	n.d.	4.20E+05	115
X18	M	36	0	4	2.75E+05	50
X19	M	58	3	4	Pos	54
X20	F	64	2	n.d.	1.11E+07	106
X21	M	65	4	1	Pos	53
X22	F	64	1	1	7.80E+05	44
X23	M	52	1	2	4.50E+06	38

Abbreviation: pos, positive: n.d., not determined within 3 months before start of therapy
